# A Self-Organized Reciprocal Decision Approach for Sensing Coverage with Multi-UAV Swarms

**DOI:** 10.3390/s18061864

**Published:** 2018-06-07

**Authors:** Runfeng Chen, Ning Xu, Jie Li

**Affiliations:** College of Mechatronics and Automation, National University of Defense Technology, Changsha 410073, China; chenrunfeng16@nudt.edu.cn (R.C.); xuning10@nudt.edu.cn (N.X.)

**Keywords:** sensing coverage, Unmanned Aerial Vehicles, cooperative motion, decentralized decision, swarm intelligence

## Abstract

This paper tackles the problem of sensing coverage for multiple Unmanned Aerial Vehicles (UAVs) with an approach that takes into account the reciprocal between neighboring UAVs to reduce the oscillation of their trajectories. The proposed reciprocal decision approach, which is performed in three steps, is self-organized, distributed and autonomous. First, in contrast to the traditional method modeled and optimized in configuration space, the sensing coverage problem is directly presented as an optimal reciprocal coverage velocity (ORCV) in velocity space that is concise and effective. Second, the ORCV is determined by adjusting the action velocity out of weak coverage velocity relative to neighboring UAVs to demonstrate that the ORCV supports a collision-avoiding assembly. Third, a corresponding random probability method is proposed for determining the optimal velocity in the ORCV. The results from the simulation indicate that the proposed method has a high coverage rate, rapid convergence rate and low deadweight loss. In addition, for up to 10^3^-size UAVs, the proposed method has excellent scalability and collision-avoiding ability.

## 1. Introduction

Sensing coverage with a UAV swarm is an important issue of how to cover an accessible region of interest (ROI) by multiple UAVs with specified sensors in an optimal manner, i.e., achieving the optimal performance including low coverage time, high coverage rate and so on. It has multifarious applications, for instance, mapping, search and rescue, forest fire monitoring and fighting, flood and earthquake response. Though ROI may vary in shape, size and may be cluttered with obstacles, sensing coverage mainly includes the following series of technical processes after obtaining surrounding information. First, the area is divided by using some diverse area decomposition method, after which the UAV makes an action decision [1,2]. Next, the UAV conducts task planning and path planning [3,4]. Last, the plan is executed by the UAV’s controller and actuator [5]. Among these technical processes, area decomposition and action decision are the most fundamental and vital; however, they are coverage decision problems in nature.

Earlier works on the coverage decision problem focused on the methods by which a single UAV covers the ROI, such as sweep manner [6,7], area decomposition [8,9] and process occasion [10]. Subsequently, researchers focused on multi-UAV cooperating coverage because of its better coverage performance than the single-UAV mode. Two methods of multi-UAV cooperating coverage are used: centralized decision and distributed decision. The former method can achieve optimal deployment and action of the UAVs based on global information; however, the expandability is limited by its exponentially increasing computation [11,12]. The latter method has more flexibility and scalability suitable for various situations since it may be difficult to achieve optimal coverage [13,14,15]. For the distributed decision, the emergence of collective behavior by simple UAVs’ interaction with local information that is self-organized, decentralized and autonomous has drawn greater attention in recent years [16]. As a result, with the development of swarm intelligence, many programs have been launched, such as SAGA, Project Wing, LOCUST, OFFSET, and Perdix, implementing the “swarm sprint”.

The various methods for swarm coverage can be primarily categorized into two strategies: partition-decision and combined-decision. Partition-decision partitions the area and then makes a decision based on the partition, whereas combined-decision makes a decision without a partition. The former strategy includes many partition methods, such as Voronoi decomposition [17], Boustrophedon decomposition [18], and Constrained Delaunay Triangulation [19], of which Voronoi decomposition, denoted as the V-based method, is the most popular method. For example, the authors in [20,21,22,23] applied the V-based method to divide the total area into several cells; subsequently, the UAVs optimized their decision based on the decomposed cells. V-based methods use the neighboring UAVs’ local positions to optimize the coverage decision but ignore the interaction between UAVs, possibly leading to oscillation or collision. Combined-decision includes the potential fields method, the neural network method, the heuristic method and the Virtual Forces algorithm. The potential fields method was used in [24]; in this method, UAVs are treated as virtual particles attracted to each other and repelled by obstacles to spread throughout the unknown environment. The neural network method in [25,26] uses a neural network to model the workspace, with robots navigating using the neural dynamics approach. The heuristic method in [27] computes the suitable placement of UAVs locally to maximize area coverage. The Virtual Forces algorithm in [28,29,30] is proposed for wireless mobile robots, which places emphasis on the biconnectivity with overlap region. Most methods are optimized in configuration space for the generated decision without considering the reciprocal of UAVs. And the optimal parameters of some models are difficult to be obtained through repeated experiments in heterogeneous swarm, especially in multi-UAVs swarm where each UAV’s sensing capability is changing with its flight height.

In this paper, a self-organized reciprocal decision approach for sensing coverage with multi-UAV swarms is proposed, whose modeling and optimization are performed in velocity space directly with no need for determining optimal parameters through repeated experiments. First, the coverage problem is directly modeled as a decision problem in velocity space because it is more flexible, scalable and immediate than modeling in configuration space. Second, the optimal reciprocal coverage-beneficial velocity (ORCV) space is determined by adjusting the action velocity out of weak coverage velocity relative to the neighboring UAVs. Moreover, it is proven that the ORCV contains the collision-avoiding assembly. Third, inspired by the Monte Carlo method, a random probability method is proposed for determining the optimal velocity in ORCV to significantly improve the effectiveness and flexibility relative to the traditional solution involving deterministic searching. During the interaction among UAVs, the covered area increases until it converges to an extremal solution. Finally, compared with two significant methods, the simulation experiments results indicate that the proposed method has higher coverage rate, faster convergence rate, and less deadweight loss than the V-based and VFA methods. In addition, for up to 10^3^-size UAVs, the proposed method is found to have excellent scalability and collision-avoiding ability. And a Robotic Operation System (ROS) Simulation is conducted to validate the proposed method.

The article is organized as follows: in Section 2, the basic idea of swarm coverage is presented. In Section 3, the reciprocal decision (RD) approach is presented, with the cooperative coverage between two-UAV proposed in Section 3.1, extending to multi-UAV swarm coverage in Section 3.2, and the constraints of avoiding collision described in Section 3.3. In Section 4, the random probability technique is proposed to determine the optimal velocity in ORCV under two different situations. The simulation and comparative analysis are shown in Section 5, and the conclusion follows in Section 6.

## 2. Basic Idea

Swarm coverage is an essential technology aiming to cover a selected region by a fleet of UAVs. It need a decision process that is self-organized, decentered and autonomous. Self-organization means that the total mission is assigned to the team with no need to decompose it into a series of subtasks and assign each UAV a specific task. Decentralization signifies the absence of a leader, with each UAV able to join or quit the team without any influence on the completion of tasks. Autonomy indicates that global behavior emerges naturally, though it is unknown to individual UAVs.

**Figure 1:**
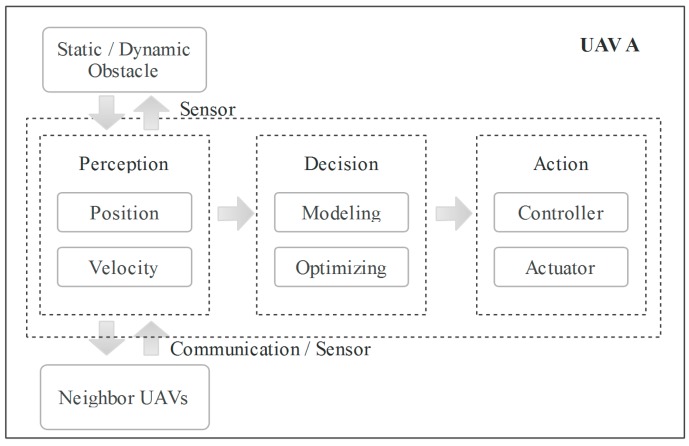
A concise overview of the continuous procedure of each UAV.

In general, the problem of swarm coverage is simplified to the process of designing a distributed algorithm for the individual UAV to cooperate with other UAVs. The general procedure of each UAV can be simply divided into three aspects—perception, decision and action—as shown in Figure 1. The first part is acquiring the information, such as position and velocity, via sensors or communication, which includes an UAV’s own information and that of other UAVs on factors such as static/dynamic obstacles and neighboring UAVs. The second part is the decision, which can be divided into two subparts: modeling and optimizing. Finally, the optimal decision will be executed by the actuator. The process is continuously repeated.

The decision part is the core of the aforementioned technique, and the present work focuses on this aspect. The reciprocal coverage method is designed to model the swarm coverage problem as the distributed optimization in velocity space by constructing the optimal region that is coverage-beneficial and collision-free. Moreover, an optimization technique is proposed to select the optimal velocity in ORCV. The processes of modeling and optimizing are presented in detail in Section 3 and Section 4, respectively.

## 3. Reciprocal Decision Approach

In this section, the reciprocal decision (RD) approach for sensing coverage, which is coverage-beneficial and collision-free, is described in detail. Section 3.1 describes the coordination between two UAVs; the coordination is extended to swarm cooperation in Section 3.2. Collision-free constraints and the relevant proof are shown in Section 3.3.

### 3.1. Two-UAV Cooperative Coverage

The region of interest (ROI) to be covered is annotated as Ω, which is a convex compact set in ${\mathbb{R}}^{2}$. A set of n UAVs share the environment, with each UAV having its shape with limited coverage. Without loss of generality, the paper assumes for simplicity that the UAVs moving in the plane ${\mathbb{R}}^{2}$ are disc shaped with radius r; the ranges of coverage and communication are discs with radius R and CR, respectively. Moreover, each UAV A has its maximum speed $v_{A}^{\max}$, maximum calculation disc-range radius $R_{A}^{\max}$, maximum calculation neighbor UAVs $n_{A}^{\max}$, and the predicted time interval τ. The position of an UAV is m, and its velocity is v. The UAVs are randomly distributed in the rectangular region $\Omega_{e}$ with both sides being $l_{e}$, and all of them are initially static.

For two UAVs A and B with limited coverage ability, they are initially close to each other, and the initial velocity $v_{A}^{cur}=v_{B}^{cur}=0$; thus, they should move by selecting their own new velocity $v_{A}^{new}$ and $v_{B}^{new}$ to maximize their own coverage, which also contributes to the total area coverage. If UAV A adopts a relative velocity to B in the time interval τ that is against to the increase of its coverage and the total coverage, we name this velocity “weak coverage velocity”. The set $WCV_{A|B}^{\tau }$ contains all weak coverage velocity for UAV A relative to UAV B, which will be formally defined in the following. Let C(m,R) be an open disc of radius R centered at m:

(1) C(m,R)={w|‖w−m‖<R}

Thus, the weak coverage velocity set $WCV_{A|B}^{\tau }$ is formally defined as follows.

(2) $$WCV_{A|B}^{\tau }=\{v|\exists t\in [0,\tau ]::tv\in C(m_{B}-m_{A},\left\|m_{B}-m_{A}\right\|)\}$$

The corresponding optimal coverage velocity set $OCV_{A|B}^{\tau }$ for UAV A relative to B is defined as follows, which is beneficial to the increase of UAV A’s coverage and the total coverage in the time interval τ:

(3) $$OCV_{A|B}^{\tau }=\{v|v\notin WCV_{A|B}^{\tau }\}$$

**Figure 2:**
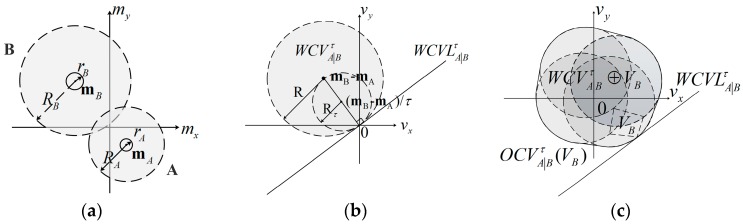
The visual display of UAVs A and B, and the weak coverage velocity $WCV_{A|B}^{\tau }$. (**a**) Display of two UAVs A and B in configuration space; (**b**) Visualization of $WCVL_{A|B}^{\tau }$ and weak coverage velocity set $WCV_{A|B}^{\tau }$ in velocity space; (**c**) Minkowski sum sets $WCV_{A|B}^{\tau }⊕V_{B}$ owing to the fluctuation of UAV B’s velocity.

The geometric interpretation of weak coverage velocity set $WCV_{A|B}^{\tau }$ for UAV A is exhibited in Figure 2; it is clear that $WCV_{A|B}^{\tau }$ and $WCV_{B|A}^{\tau }$ are symmetric with the origin. In Figure 2a, a visual display of two UAVs A and B in the configuration space is shown, while UAVs A and B have different radius of shapes ($r_{A}$ and $r_{B}$) and communication ($R_{A}$ and $R_{B}$), which are centered at $m_{A}$ and $m_{B}$. In Figure 2b, the weak coverage velocity set $WCV_{A|B}^{\tau }$ (gray) for UAV A is presented in velocity space as a circle with the disc of radius $R_{\tau }=\left\|m_{B}-m_{A}\right\|/\tau$ centered at $(m_{B}-m_{A})/\tau$, where τ is the predicted time interval; here, τ=1 and τ=2. $WCVL_{A|B}^{\tau }$ is a line that separates the weak coverage velocity set $WCV_{A|B}^{\tau }$ and the optimal coverage velocity set $OCV_{A|B}^{\tau }$. It is clear that an UAV is difficult to move at a fixed velocity $v_{B}$ in time interval τ. Thus, if $v_{B}\in V_{B}$ ($V_{B}$ is a scope that includes UAV B’s all possible velocity $v_{B}$ in the time interval τ), then UAV A should select a velocity that is out of the Minkowski sum [31] sets $WCV_{A|B}^{\tau }⊕V_{B}$ to increase its area coverage and the total coverage, as shown in Figure 2c. And the optimal coverage velocity $OCV_{A|B}^{\tau }(V_{B})$ is defined as follows:

(4) $$OCV_{A|B}^{\tau }(V_{B})=\{v|v\notin WCV_{A|B}^{\tau }⊕V_{B}\}$$

Considering the reciprocal of UAVs, if a pair of velocity sets $V_{A}$, $V_{B}$ for UAV A and B respectively satisfy the constraints $V_{A}\subseteq OCV_{A|B}^{\tau }(V_{B})$ and $V_{B}\subseteq OCV_{B|A}^{\tau }(V_{A})$, then they are reciprocally coverage-beneficial. $ORCV_{A|B}^{\tau }$ and $ORCV_{B|A}^{\tau }$ are the sets that are reciprocal coverage-beneficial and maximal during the time interval τ, while they have the most velocities close to A and B’s current velocities $v_{A}^{cur}$ and $v_{B}^{cur}$. The sets of $ORCV_{A|B}^{\tau }$ and $ORCV_{B|A}^{\tau }$ are defined as follows:

**Definition** **1.** *(optimal reciprocal coverage-beneficial velocity).*$\bar{\bar{A}}$*is the cardinality of the set*A*[32].*$ORCV_{A|B}^{\tau }$*and*$ORCV_{B|A}^{\tau }$*are defined as*$OCV_{A|B}^{\tau }(ORCV_{B|A}^{\tau })=ORCV_{A|B}^{\tau }$*and*$OCV_{B|A}^{\tau }(ORCV_{A|B}^{\tau })=ORCV_{B|A}^{\tau }$. *For all other pairs of reciprocal coverage-beneficial velocities sets*$V_{A}$*and*$V_{B}$*(i.e.,*$V_{A}\subseteq OCV_{A|B}^{\tau }(V_{B})$*and*$V_{B}\subseteq OCV_{B|A}^{\tau }(V_{A})$*), and for all radii*r*, it holds that:*

(5) $$\left\{ \begin{array}{l} S_{A}=ORCV_{A|B}^{\tau }\cap C(v_{A}^{opt},r)\\ S_{B}=ORCV_{B|A}^{\tau }\cap C(v_{A}^{opt},r)\\ {S_{A}}^{\prime }=V_{A}\cap C(v_{A}^{opt},r)\\ {S_{B}}^{\prime }=V_{B}\cap C(v_{B}^{opt},r)\\ S_{\min}=\min(\bar{\bar{{S_{A}}^{\prime }}},\bar{\bar{{S_{B}}^{\prime }}})\\ \bar{\bar{S_{A}}}=\bar{\bar{S_{B}}}\\ \bar{\bar{S_{A}}},\bar{\bar{S_{B}}}\geq S_{\min} \end{array}\right.$$

**Figure 3:**
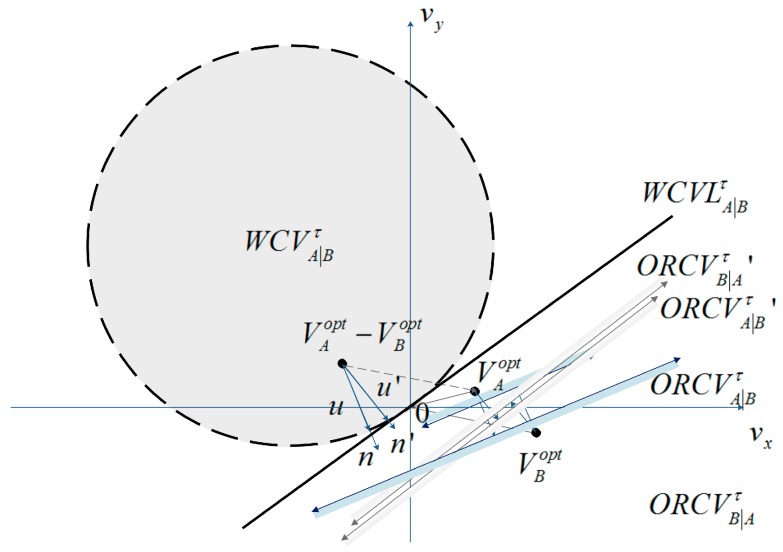
Two approaches of describing $ORCV_{A|B}^{\tau }$ for UAV A relative to B.

In other words, $ORCV_{A|B}^{\tau }$ and $ORCV_{B|A}^{\tau }$ contain the most velocities close to UAV A and B’s current velocities $v_{A}^{cur}$ and $v_{B}^{cur}$. The difference between the velocities and $v_{A}^{cur}$ and $v_{B}^{cur}$ is equal for A and B. The establishment of $ORCV_{A|B}^{\tau }$ and $ORCV_{B|A}^{\tau }$ is shown geometrically in Figure 3.

$v_{A}^{cur}-v_{B}^{cur}\in WCV_{A|B}^{\tau }$ means that it will lead to the weak coverage for UAV A and B. In such situation, if A and B adopt new velocity $v_{A}^{new}$ and $v_{B}^{new}$ respectively, which satisfies that $v_{A}^{new}-v_{B}^{new}$ is out of $WCV_{A|B}^{\tau }$, then the total coverage area will be increased. Let u be the vector from $v_{A}^{cur}-v_{B}^{cur}$ to the closest point on the boundary of $WCV_{A|B}^{\tau }$:

(6) $$u=(\underset{v\in \partial WCV_{A|B}^{\tau }}{\arg\min}\left\|v-(v_{A}^{cur}-v_{B}^{cur})\right\|)-(v_{A}^{cur}-v_{B}^{cur})$$

Alternatively, let $u^{\prime }$ be the vector from $v_{A}^{cur}-v_{B}^{cur}$ to the closest point on the boundary of $WCVL_{A|B}^{\tau }$:

(7) $$u^{\prime }=(\underset{v\in WCVL_{A|B}^{\tau }}{\arg\min}\left\|v-(v_{A}^{cur}-v_{B}^{cur})\right\|)-(v_{A}^{cur}-v_{B}^{cur})$$

n is the outward normal vector of the boundary of $WCV_{A|B}^{\tau }$ at point $(v_{A}^{cur}-v_{B}^{cur})+u$. Because u is the smallest change required for the relative velocity of UAV A and B to avert weak coverage within τ time and both UAVs share the responsibility of avoiding weak coverage, UAV A adapts velocity 0.5u at least, and B is responsible for another half:

(8) $$ORCV_{A|B}^{\tau }=\{v|(v-(v_{A}^{cur}+\frac{1}{2}u))\cdot n\geq 0\}$$

Clearly, the set $ORCV_{B|A}^{\tau }$ for B is defined symmetrically. Moreover, the above method is applicable when $v_{A}^{cur}-v_{B}^{cur}\notin WCV_{A|B}^{\tau }$, which indicates that A and B will not lead to the weak coverage if they still adopt their current velocities $v_{A}^{cur}$ and $v_{B}^{cur}$, respectively. However, in this situation, both UAVs can also utilize the abovementioned method to maintain a coverage-beneficial movement.

### 3.2. Multi-UAV Swarm Coverage

**Figure 4:**
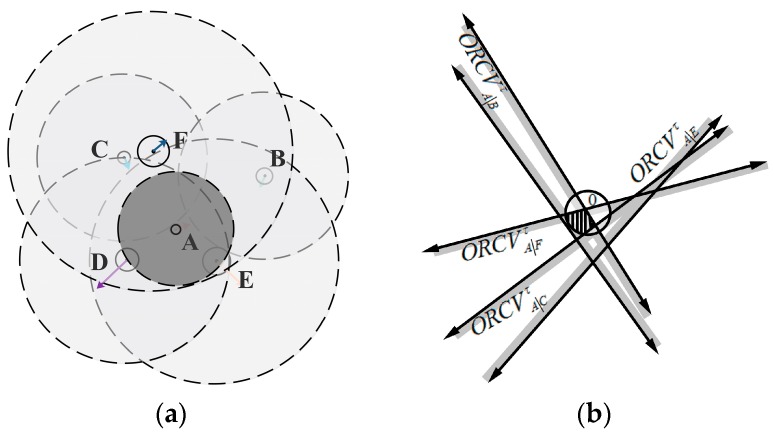
The construction of optimal velocity space. (**a**) Visual display of UAVs’ situation; (**b**) The geometrical expression of $ORCV_{A}^{\tau }$.

The overall method is as follows: UAV A executes a continuous cycle of sensing and acting with time step Δt. In each period, UAV A acquires the coverage radius, current positions and velocities of its neighboring UAVs and itself. Let $D_{A}^{\max}$ be the maximal calculation distance of UAV A with respect to its neighboring UAV. $n_{A}^{\max}$ ($n_{A}^{\max}\in \mathbb{N}$) is a positive constant of the maximum considered neighboring number for UAV A. Hence, when any UAV B satisfies the constraints $\left\|m_{A}-m_{B}\right\|\leq D_{A}^{\max}$, UAV A only concerns its $n_{A}^{\max}$ neighboring UAV B that are closer than the others in Euclidean distance. KD-tree is used for UAV A to search the neighboring UAVs in this paper. UAV A deduces the optimal half-plane of velocities $ORCV_{A|B}^{\tau }$ relative to neighboring UAVs B. And $ORCV_{A}^{\tau }$ is a set of optimal velocity spaces that are optimal for UAV A relative to all its $n_{A}^{\max}$ neighboring UAVs, which is the intersection of the half-planes of optimal velocities conducted by its neighbor. UAV A is also conditioned to its own maximum speed $v_{A}^{\max}$. Therefore, the optimal velocity set $ORCV_{A}^{\tau }$ for UAV A is defined as follows, and its geometrical expression is shown in Figure 4:

(9) $$ORCV_{A}^{\tau }=C(0,v_{A}^{\max})\cap \underset{B\neq A}{\cap }ORCV_{A|B}^{\tau }$$

Next, UAV A selects a new velocity $v_{A}^{new}$ for itself that is optimal (center velocity of the optimal space in this paper) among all velocities within the optimal velocities space:

(10) $$v_{A}^{new}=\underset{v\in ORCV_{A}^{\tau }}{\arg\min}\left\|v-v_{A}^{opt}\right\|$$

Finally, the UAV A reaches its new position:

(11) $$m_{A}^{new}=m_{A}+v_{A}^{new}\Delta t$$

Equations (9) and (10) are the critical computation of $v_{A}^{new}$, which can be done by linear programing effectively. Though $v_{A}^{new}$ is limited to the maximum velocity of UAV A, it does not change the algorithm dramatically. $ORCV_{A}^{\tau }$ is a convex region bounded by linear constraints, which is added by random order in the effective algorithm [33]. Therefore, the running time of algorithm still depends on constraints’ number n, which is equal to $n_{A}^{\max}$ here. It has an expected running time of O(n).

**Figure 5:**
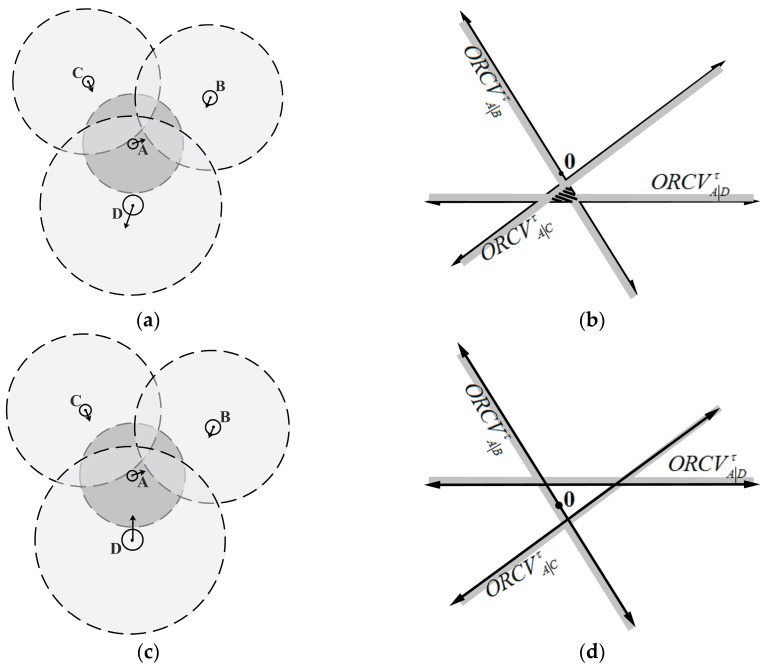
Two situations of optimal velocity space. (**a**) Available situation; (**b**) Available space; (**c**) Vacant situation; (**d**) Vacant space.

$ORCV_{A}^{\tau }$ may be available or vacant (shown as Figure 5), which will adopt different optimization strategies. In the Section 5, the random probability method will be utilized for searching the optimal velocity.

### 3.3. Collision-Free Constrains

#### 3.3.1. Collision Avoidance between UAVs

The ORCV described by the RD method as noted above satisfies the constraints of avoiding collision with other UAVs, as will be proved as below.

It is clear that UAVs A and B will collide within τ time if UAV A selects the velocity relative to B within $VO_{A|B}^{\tau }$ [34] defined as follows:

(12) $$VO_{A|B}^{\tau }=\{v|\exists t\in [0,\tau ]::tv\in C(m_{B}-m_{A},r_{A}+r_{B})\}$$

Obviously, although UAVs remain in a crowded environment, they are assumed to be initially collision-free. Next, the UAVs can move without collision between other UAVs by employing the RD method. The proof is shown as follows:

**Corollary** **1.** *For any time interval* τ *, it holds that:*

(13) $$VO_{A|B}^{\tau }\subseteq WCV_{A|B}^{\tau }$$

**Proof.**  For any time interval τ, UAV A and B remain in a crowded environment but without collision (see Figure 2a) at the beginning, which satisfies:

$$\forall t\in [0,\tau ]::\left\|m_{B}-m_{A}\right\|\geq r_{A}+r_{B}$$

Then:

$$\forall t\in [0,\tau ]::C(m_{B}-m_{A},r_{A}+r_{B})\subset C(m_{B}-m_{A},\left\|m_{B}-m_{A}\right\|)$$

Furthermore:

$$VO_{A|B}^{\tau }=\{v|\exists t\in [0,\tau ]::tv\in C(m_{B}-m_{A},r_{A}+r_{B})\}$$

Moveover:

$$WCV_{A|B}^{\tau }=\{v|\exists t\in [0,\tau ]::tv\in C(m_{B}-m_{A},\left\|m_{B}-m_{A}\right\|)\}$$

Thus:

$$VO_{A|B}^{\tau }\subset WCV_{A|B}^{\tau }$$

□

**Figure 6:**
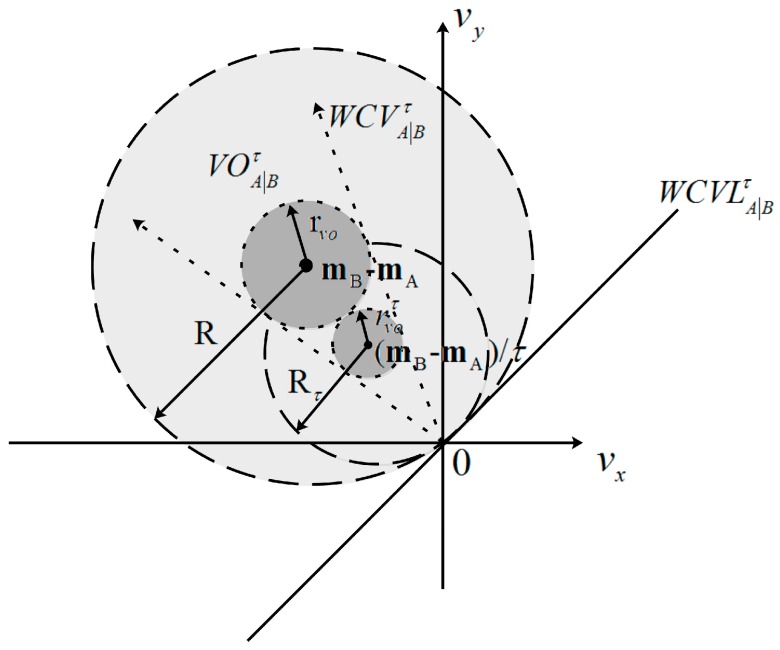
The geometrical expression of Corollary 1.

Corollary 1 is directly shown in the geometry in Figure 6; the space of $WCV_{A|B}^{\tau }$ always contains $VO_{A|B}^{\tau }$ at any time τ, and the $VO_{A|B}^{\tau }$ is always to the left of $WCVL_{A|B}^{\tau }$, which indicates that coverage-beneficial velocity will not collide in the time window τ.

#### 3.3.2. Avoiding Collision with Obstacles

The coverage environment contains not only UAVs but also static obstacles and perhaps unknown dynamic objects that are regarded as dynamic obstacles. The reciprocal coverage method is flexible and extensible and can easily add the constraints of collision-free to shrink the ORCV to meet the need.

##### Static Obstacle

UAVs should take full responsibility for coverage-beneficial motion when faced with static obstacles, resulting from the fact that the obstacle cannot cooperate.

**Figure 7:**
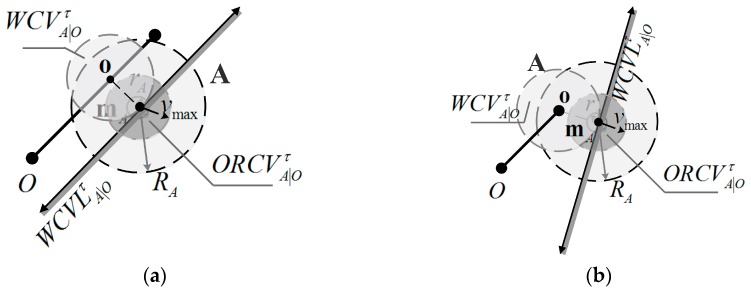
Two cases of visual display of UAV A, obstacle O and $ORCV_{A|O}^{\tau }$. (**a**) The most weak coverage point o is on the edge of the segment O; (**b**) The most weak coverage point o is the endpoint of the segment O.

In this paper, the obstacles are modeled as a collection of line segments. Let O be one of these line segments, and let A be an UAV with shape-radius $r_{A}$ and coverage-radius $R_{A}$ positioned at $m_{A}$. Next, the weak coverage velocity set $WCV_{A|O}^{\tau }$ generated by obstacle O is defined as follows (o is a selected point as shown in Figure 7):

(14) $$WCV_{A|O}^{\tau }=\{v|\exists t\in [0,\tau ]::tv\in C(o-m_{A},\left\|o-m_{A}\right\|)\}$$

When the distance between UAV A and obstacle O is less than a constant $\lambda_{A}$, UAV A should consider the obstacle O. If allowing UAVs not to be sensitive to the obstacle, then $\lambda_{A}$ can be less than coverage-radius $R_{A}$. Otherwise, $\lambda_{A}$ should be equal to $R_{A}$. If UAV A’s velocity $v_{A}$ is within $WCV_{A|O}^{\tau }$, then the weak coverage during the time interval τ is appeared relative to obstacle O. $ORCV_{A|O}^{\tau }$ is defined for the optimal velocity to realize the coverage-beneficial motion relative to obstacle O, which is the intersection of $WCVL_{A|O}^{\tau }$ and $C(0,v_{\max})$. $WCVL_{A|O}^{\tau }$ is determined by selected point o in segment O, which is the weakest coverage point, as shown in Figure 7a,b.

The visual displays of UAV A, obstacle O and $ORCV_{A|O}^{\tau }$ are shown in Figure 7. Figure 7a is a case of UAV A and line-segment obstacle O, where the selected point o is the point with $om_{A}⊥O$ in segment O. Figure 7b is another case in which o is the endpoint of O, which is close to $m_{A}$. The geometric construction of the coverage-beneficial and collision-free space $ORCV_{A|O}^{\tau }$ with the limit of maximum speed $v_{\max}$ is shown. In this paper, UAV A will not consider obstacle O that is out of range when the distance between UAV A and obstacle O is greater than their collision distance value $R_{A}$.

##### Dynamic Obstacle

The crux is that dynamic obstacles do not coordinate and even interfere with the coverage, in contrast with the UAVs. Therefore, the UAVs should take full responsibility for coverage-beneficial motion.

As discussed in Section 3.1, u is the smallest change required to the relative velocity of A and B to avoid weak coverage within time τ, but in contrast to Section 3.1, UAV A should take full responsibility for collision-free motion or even more, UAV A adapts its velocity by αu (α≥1). The constraints are as follows:

(15) $$ORCV_{A|B}^{\tau }=\{v|(v-(v_{A}^{cur}+\alpha u))\cdot n\geq 0\},\alpha \geq 1$$

## 4. Optimal Velocity Decision

The ORCV for UAV A is constructed in Section 4. In this section, a technique for searching the optimal velocity in ORCV is declared formally, which is effective relative to other traditional traversal methods.

### 4.1. Random Probability Method

The traditional traversal method must confirm the exact region of the search space; however, the exact space is typically difficult to obtain because of the uncertainty of shape. It is inefficient to traverse all the possible values of ORCV. Therefore, a random probability method inspired by the Monte Carlo method is proposed for identifying the optimal velocity within the confirmed optimal space.

The random probability method utilizes the concept of convergence in probability, where the mean of abundant random optimal velocities will approach the center of the optimal velocity space, despite the specific shape of ORCV being unknown. The core of the random probability method is shown as follows:

**Corollary** **2.** *For the set of* G *composed of abundant random velocities* $v_{rand}$ *,* $v_{rand}\in PR$ *, it holds that:*

(16) $$AVE(G)\to v_{opt}$$

**Proof.**  Assuming all the velocities of PR are traversed, the following is obtained:

$$v_{opt}=\sum v_{i}/Num(PR),v_{i}\in PR$$

while:

$$AVE(G)=\sum_{i=1}^{n}v_{i}/n,v_{i}\in G$$

When n is sufficiently large, according to the Bernoulli law of large numbers:

$$\underset{n\to \infty }{\lim}p\{\left|AVE(G)-v_{opt}\right|<\varepsilon \}=1,\forall \varepsilon >0$$

□

The symbols used in this paper are defined in Table 1.

### 4.2. Optimum Available

When the optimal region is available, the exploration of optimal velocity follows Algorithm 1. Algorithm 1 is an effective technique to determine an optimal velocity resolution that is very close to the center of ORCV. Algorithm 1 is described below.
**Algorithm 1.** Random Probability Exploration of the Optimal Velocity $v_{A}^{opt}$.**Input:** UAV A maximal velocity $v_{A}^{\max}$, constrains of neighbor UAVs $ORCV_{A|*}^{\tau }$
**Output:** The optimal velocity decision $v_{A}^{opt}$
1: Computational Rectangle Domain: $RandVelRange=Square(\left\|v_{A}^{\max}\right\|)$
2: Random Velocity: $v_{rand}=RV(RandVelRange)$
3: Set the Accuracy: AN=1000
4: Initialization: N=1, FN=0, FD=Φ
5: **while**
N≤AN
**do**
6:  **if**
$\left\|v_{rand}\right\|\leq \left\|v_{A}^{\max}\right\|$
**then**
7:   **if**
$v_{rand}\subset ORCV_{A|*}^{\tau }$
**then**
8:    $v_{rand}\to FD$
9:    FN=FN+1
10:  **end if**
11: **end if**
12: N=N+1
13: **end while**
14: Output $v_{A}^{opt}=AVE(FD)$  ⊳ The optimal velocity has been explored. 15: **return**.

### 4.3. Vacant Optimal Velocity Space

When the ORCV is empty, the exploration of optimal velocity follows Algorithm 2 as below. Algorithm 2 is an effective technique to confirm the likelihood that the ORCV is vacant.
**Algorithm 2.** Lounger strategy.**Input:** UAV A maximal velocity $v_{A}^{\max}$, constrains of neighbor UAVs $ORCV_{A|*}^{\tau }$
**Output:** The optimal velocity decision $v_{A}^{opt}$
Process 1~13 is same as **Algorithm 1**
14: **if**
FN=0
15: Output $v_{A}^{opt}=IdleVel()$
⊳ Adopt idle velocity. 16: **end if**
17: **return**.

### 4.4. Numerical Test

The ORCV constructed via the RD method may be available or null. Therefore, the numerical test is conducted in these two situations to verify the feasibility and rationality of the technique and the value adopted in this paper.

#### 4.4.1. Available Set

**Figure 8:**
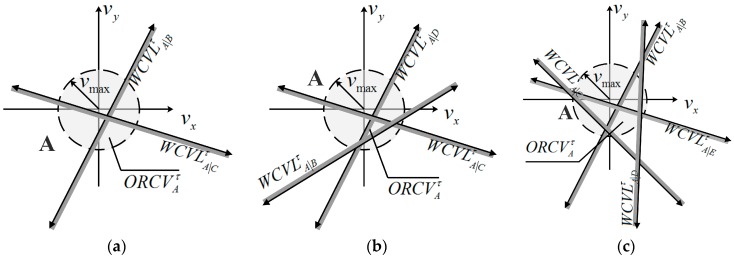
Three representative cases of optimization set. (**a**) ORCV is a shape of sector; (**b**) ORCV is a shape of triangle; (**c**) ORCV is an irregular shape.

When the ORCV is available, it may present various situations, such as circular sector, regular polygon and irregular shape (see Figure 8).

In this paper, the center of the ORCV for each UAV is regarded as the optimal choice because of the equilibrium of benefit relative to each other neighbor. However, it is difficult to obtain the optimum with calculations because the shape of the ORCV is difficult to determine. Thus, a technique for selecting a velocity as close as possible to the optimal velocity is proposed.

**Figure 9:**
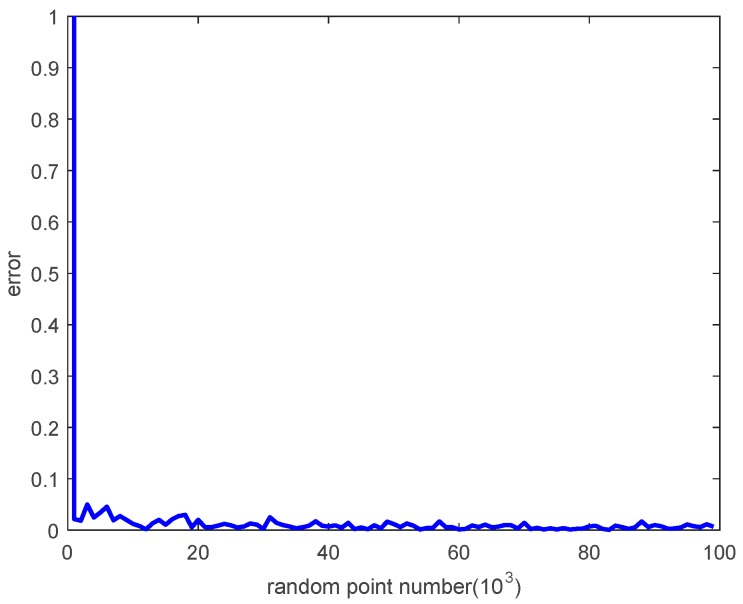
The error between the generated velocity and the optimal velocity.

The error between the velocity generated by the proposed technique and the optimal velocity is shown in Figure 9.

It can thus be seen that the error decreases as the number of random points increases, and it is very close to zero with a large number of random points.

#### 4.4.2. Null Set

**Figure 10:**
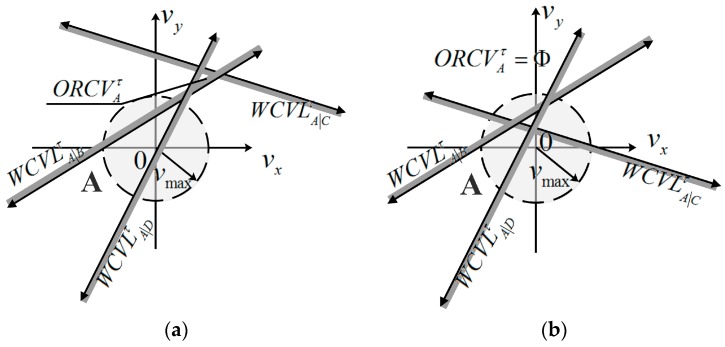
Two situations of null set. (**a**) $ORCV_{A}^{\tau }\cap C(0,v_{\max})=\Phi$; (**b**) $ORCV_{A}^{\tau }=\Phi$.

The situation of a null set may occur in the construction of the ORCV, such as the case of overspeed or excess constraints (see Figure 10). However, it is also difficult to determine whether the ORCV is empty. Therefore, the proposed technique is suitable to such a case.

**Figure 11:**
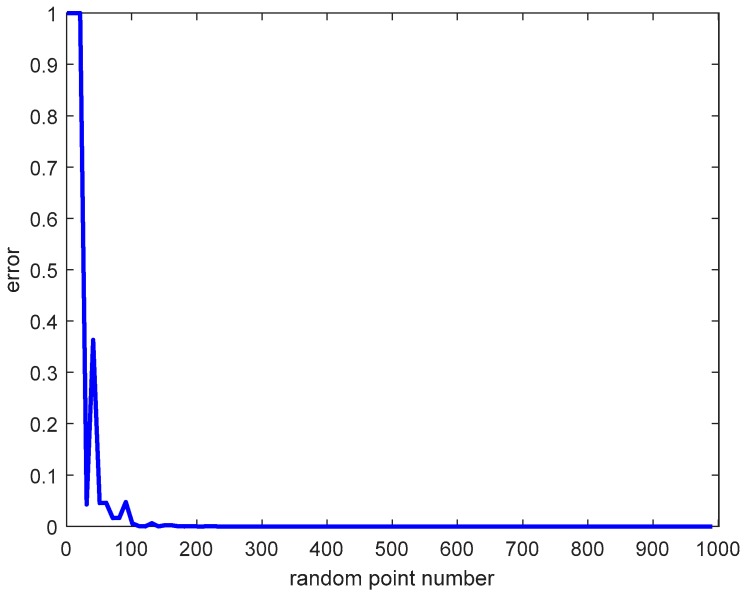
The probability of misjudgment of the null set with the number of random points.

The error of evaluation of the null set while the ORCV is non-empty is shown in Figure 11. It is intuitive that the error declines with the augmentation of the number of random points.

The error nearly reaches zero after 200 random points, which confirms the validity of the proposed technique to evaluate the null set when the number of random point is set to 1000.

The optimal velocity can be improved with an increased number of random points with a small penalty. However, it is sufficient to set the number of random points to 1000 in this paper.

## 5. Simulation and Results

To validate the effectiveness and performance of the proposed method with or without static obstacles, small-scale coverage is simulated in Section 5.1 and extended to large scale in Section 5.2, which includes various performances, such as coverage rate, deadweight loss, trajectory smoothness, and convergence speed. In addition, a Robotic Operation System (ROS) simulation is conducted to improve the reliability of the proposed method further. The simulation is programmed in C++ using OpenMP to parallelize key computation across eight Intel(R) 2.60 GHz cores. The simulations parameters are shown in Table 2.

The algorithm will be terminated when $\sup{\left\|a_{i+1}^{*}-a_{i}^{*}\right\|}_{2}\leq \zeta$. UAVs are random distributed in $\Omega_{a}$ in the beginning.

### 5.1. Small-Scale

**Figure 12:**
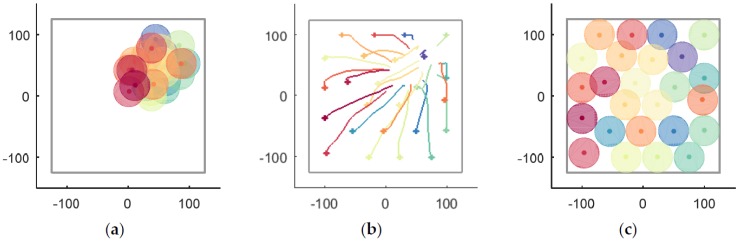
The entire process of UAVs’ coverage in a closed environment $\Omega_{e}$ without obstacles. (**a**) the initial positions; (**b**) the moving trajectories; (**c**) the optimal coverage.

A case in a closed environment $\Omega_{e}$ without obstacles is shown in Figure 12. First, UAVs are randomly distributed in crowded region $\Omega_{a}$, as shown in Figure 12a. Thus, according to UAVs’ local information, they begin to disperse to improve coverage without collision. The UAVs’ moving trajectories are recorded in Figure 12b, where the smoothness of the trajectories is noticeable. The algorithm is convergent when simulation step k=574, and the optimal coverage position of each UAV is shown in Figure 12c.

**Figure 13:**
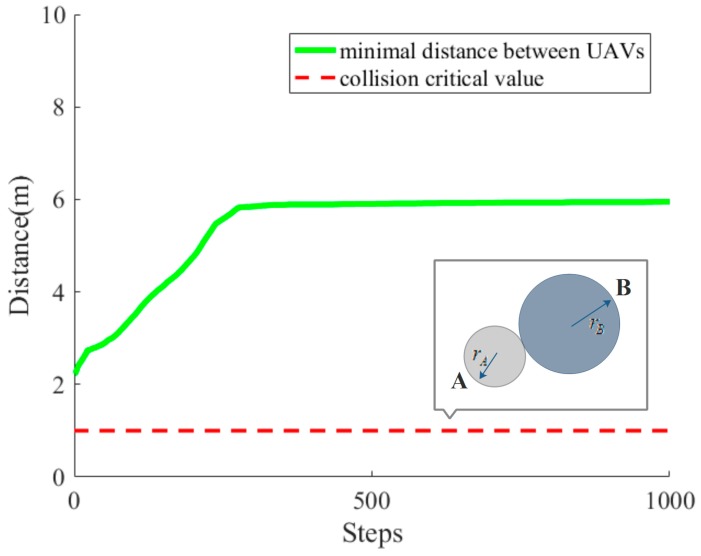
The variation of the minimum distance between UAVs.

During the movement of UAVs, collisions with other UAVs are avoided. The minimum distance between UAVs (green thick line) and the collision critical value (red thin dashed line) in each simulation step k are shown in Figure 13, which demonstrates the collision-free movement of UAVs intuitively.

**Figure 14:**
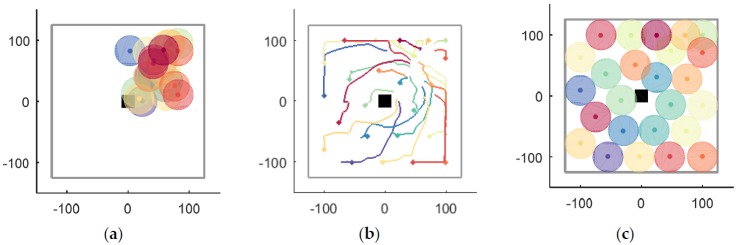
The process of coverage with obstacle $\Omega_{o}$. (**a**) the initial positions; (**b**) The moving trajectories; (**c**) The optimal coverage.

A case with a rectangular static obstacle $\Omega_{o}$ with both sides being 10 m as shown in Figure 14 is considered, while other conditions are same as before. UAVs’ initial positions are shown in Figure 14a. Next, UAVs begin to disperse to improve coverage, balancing the avoidance of collision with other UAVs and static obstacles. The obstacle is considered only when it is within the range of UAV A, which is equal to coverage radius R in this paper. UAVs’ moving trajectories are recorded in Figure 14b, where circumnavigation around the obstacle is noticeable. The algorithm is convergent when the simulation step k=1248 and the optimal coverage position of each UAV is shown in Figure 14c.

**Figure 15:**
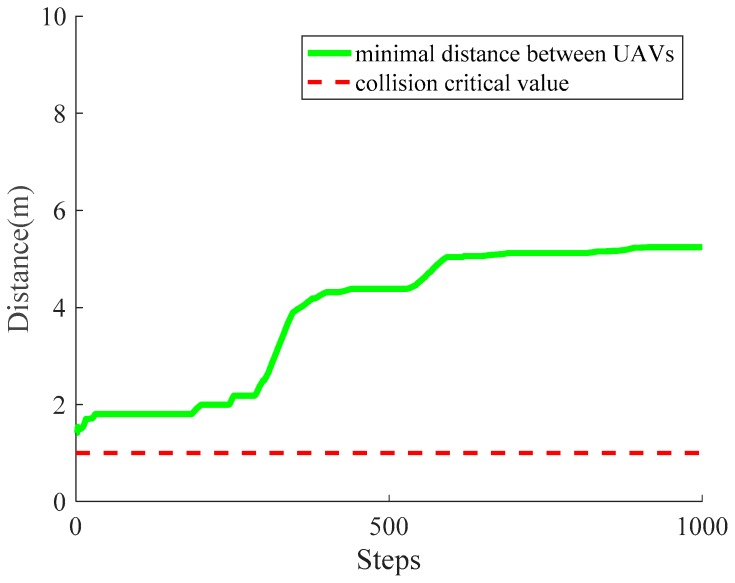
The variation of the minimum distance between UAVs.

During the movement of UAVs, collisions with other UAVs and with obstacles are avoided. If the minimal distance between two UAVs is greater than the collision critical value (the sum of two UAVs’ shape radii), then the collision between these two UAVs would occur at that moment. In Figure 15, the minimum distance between UAVs (green thick line) and the collision critical value (red thin dashed line) in each simulation step k are shown, demonstrating the effectiveness of averting collision with other UAVs intuitively.

**Figure 16:**
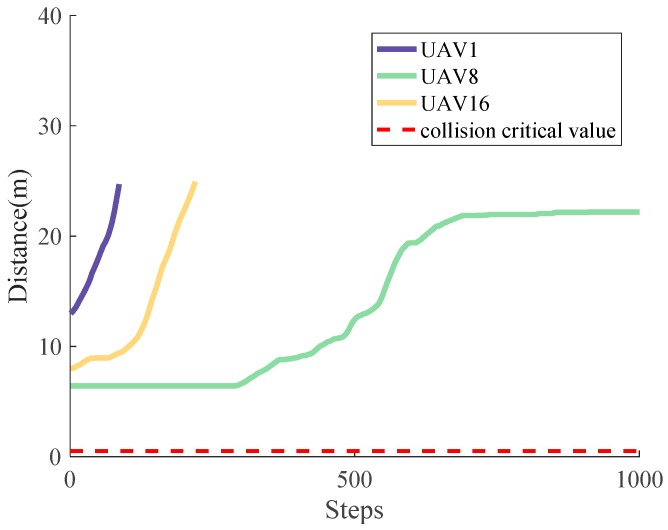
The variation of distance between UAVs and obstacles.

In Figure 16, the distance between the UAVs and obstacle is shown only when the obstacle is within the range of the UAV, demonstrating the effectiveness of averting collision with an obstacle intuitively. In this case, only UAV 1, UAV 8 and UAV 16 are assumed to consider the obstacle, while other UAVs only require consideration of their neighboring UAVs and the boundary of $\Omega_{e}$.

To quantify and objectively appraise the performance of the RD method proposed in this paper, comparisons with the traditional V-based method [20] and the VFA method [30] under the environment without obstacles is shown as follows. The traditional V-based method and the VFA method use the same parameters as the RD method.

**Figure 17:**
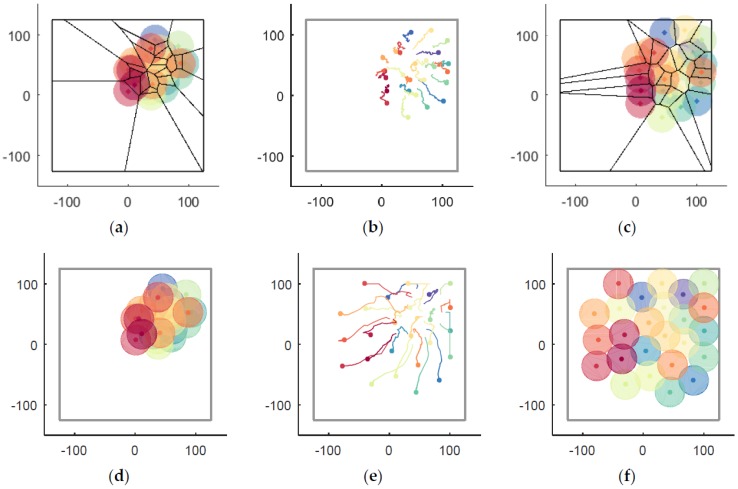
The coverage situations of the V-based and VFA methods. (**a**) The V-based coverage (k=0); (**b**) The recorded trajectories of UAVs by the V-based method (k=0~574); (**c**) The V-based coverage at k=574; (**d**) The VFA coverage (k=0); (**e**) The recorded trajectories of UAVs by the VFA method (k=0~574); (**f**) The VFA coverage at k=574.

The coverage situations of the V-based method and the VFA method are shown in Figure 17, of which (a–c) are belong to the V-based method and (d–f) are belong to the VFA method. The initial positions of each UAV are shown in Figure 17a,d, the recorded trajectories of UAVs in simulation step k=0~574 are shown in Figure 17b,e, and the situation at k=574 are displayed in Figure 17c,f, where the RD method (shown in Figure 12c) is superior to the V-based method and the VFA method in the field of convergence speed and the coverage rate can be easily found visually.

**Figure 18:**
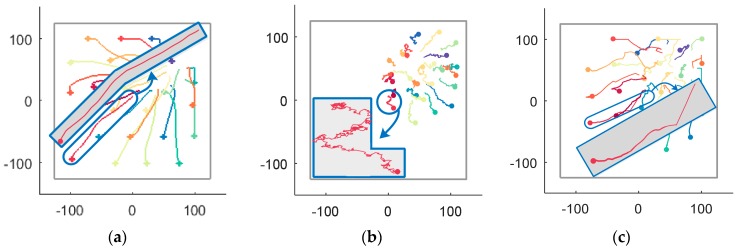
The recorded trajectories of UAVs generated by the simulation step k=0~574 by the RD, V-based and VFA methods. (**a**) The RD method’s trajectories; (**b**) The V-based method’s trajectories; (**c**) The VFA method’s trajectories.

During the simulation step k=0~574, the trajectories of UAVs generated by the RD method, the V-based method and the VFA method are shown in Figure 18, where Figure 18a is the RD method’s trajectories, Figure 18b is the V-based method’s trajectories and Figure 18c is the VFA method’s trajectories. A feature of the trajectory of UAV 23 reveals the improved smoothness and lower oscillation of the RD method than the V-based method and the VFA method. The reason is that RD considers the reciprocal of UAVs but the other methods ignore it.

**Figure 19:**
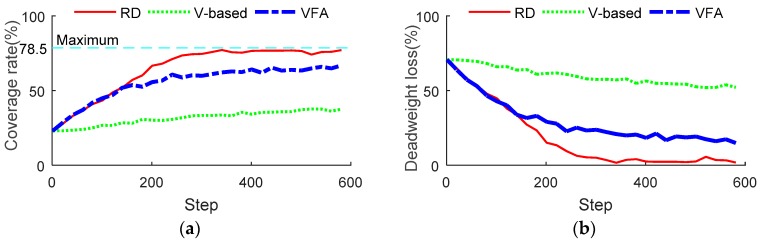
The comparison of coverage rate and deadweight loss. (**a**) Comparison of coverage rate; (**b**) Comparison of deadweight loss.

Next, the comparison of the coverage rate and deadweight loss among the RD, V-based and VFA methods are exhibited in Figure 19a,b respectively, which shows that the RD method has a higher coverage rate and less deadweight loss than the other two methods at the same time. The advantage of RD in coverage rate and deadweight loss also owes to its consideration of the UAVs’ reciprocity.

**Figure 20:**
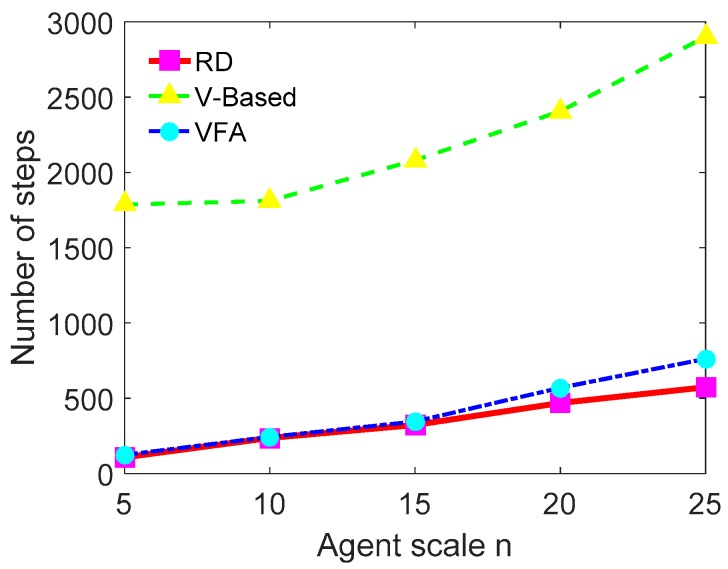
The comparison of convergence speed in different scale of UAVs.

With the increasing scale of UAVs, the difference in convergence speed among these three methods is shown in Figure 20, which indicates that the RD method is more scalable and adaptable than the other two methods.

The calculation speed in various environments but with n=25 UAVs is shown in Table 3. For each UAV, it takes 14.807 ms in average to optimize coverage decision while utilizing the RD method. For each UAV, more than 500 ms is required to make a decision using the V-based method and the VFA method needs about 42.798 ms in average. This is because RD is direct optimized in velocity space while V-based method spends a lot of time in Voronoi partition in configuration space.

### 5.2. Large-Scale

**Figure 21:**
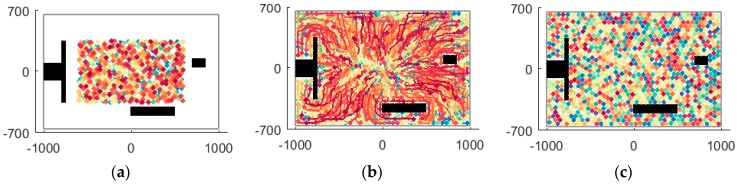
Cooperation coverage by 1000 UAVs. (**a**) Coverage (t=0); (**b**) Trajectory (t=0~5226); (**c**) Coverage (t=5226).

To verify the scalability of the RD method, a case of swarm coverage is simulated by 1000 UAVs in a 2000 m×1000 m rectangular region ${\Omega_{C}}^{\prime }$ with static obstacles as shown in Figure 21. The parameter of each UAV is the same as in Section 5.1.

First, UAVs are static and randomly distributed within a 1000 m×500 m rectangular region ${\Omega_{a}}^{\prime }$ as shown in Figure 21a. During the collision-free interaction among UAVs, the covered area is increasing, as shown in Figure 21b. Finally, the algorithm is converged to an extremum solution, as shown in Figure 21c.

From the simulation and data above, the advantages of RD can be easily summarized. First, RD with the property of distributed, asynchronous and self-organized UAVs has a higher coverage rate and less deadweight loss while converging quickly. Additionally, RD leads to smoother moving trajectory and faster decisions. Finally, the RD method is more adaptive to various scenes, such as situations with obstacles or large-scale coverage, and provides the capacity for collision-avoiding, scalability and flexibility.

### 5.3. Robotic Operation System (ROS) Simulation

For the sake of verifying the proposed method’s effectiveness further, a simulation of multi-UAV sensing coverage is conducted by using ROS Jade and Gazebo 5.0 on an Intel PC (×86) running Ubuntu 14.04.

**Figure 22:**
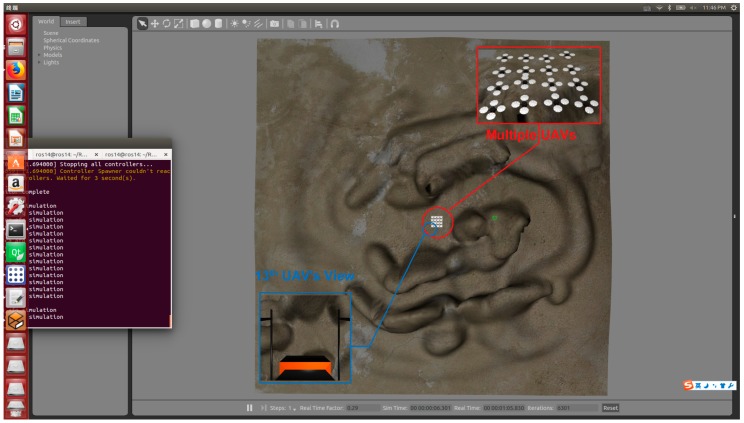
A screenshot of the simulation on Gazebo.

Limited by PC’s performance, a mimitype multi-UAV sensing coverage is customized, where 16 UAVs execute a cooperative coverage of mountainous region. The region is an area of 16,384 square meters, whose both length and width are 128 m. Each UAV flies at 50-m height with a maximum velocity of 20 m/s and sensing scope of 30 m×30 m. In addition, each UAV is instantiated as an independent ROS node, which means that the simulation is running in a distributed way. The printscreen of simulation on Gazebo is exhibited in Figure 22.

**Figure 23:**
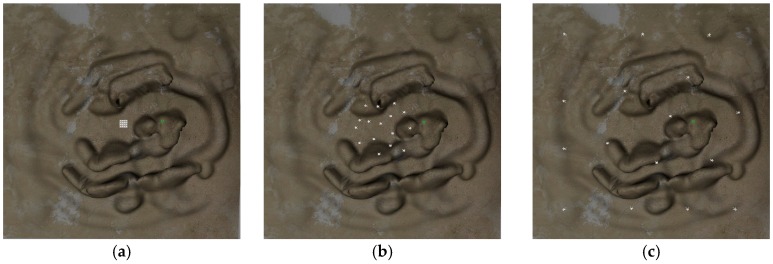
Three typical moments of multi-UAVs sensing coverage: (**a**) Initial coverage (t=0); (**b**) Scatter moment (t=2 s); (**c**) Steady-state coverage (t=10 s).

In Figure 23, three typical moments are captured, where Figure 23a shows that 16 UAVs assemble in the center of the mountainous region at t=0. Then, UAVs begin to scatter for maximizing the sensing coverage in Figure 23b. Finally, UAVs reach steady state that they have get their maximum coverage at 10 s. As can be seen from the simulation, the proposed method has potential and practical value.

## 6. Conclusions and Future Work

In this paper, a reciprocal decision approach is proposed for sensing coverage with multi-UAV swarms. The approach is self-organized, distributed, and autonomous, with no need for determining optimal parameters through repeated experiments, which is more suitable for heterogeneous sensing coverage especially in multi-UAVs swarms where each UAVs’ sensing capability is changing with its flight height. In contrast to the traditional configuration methods, the coverage problem is directly optimized in velocity space, which is more concise and efficient. First, the reciprocal of UAVs has been considered to reduce the oscillation of UAVs’ trajectories. Second, the coverage-beneficial and collision-free set ORCV is determined by adjusting the velocity out of WCV relative to neighboring UAVs. Furthermore, a corresponding random probability method is proposed for selecting the optimal velocity in ORCV. Finally, compared with two significant methods, the simulation results corroborate that the proposed method has better performance in terms of coverage rate, convergence rate, trajectory smoothness and scalability than the V-based and VFA methods. In addition, a ROS simulation is conducted to validate the availability and practicability of the RD method. The model of UAVs can be more specific in terms of the kinematics and dynamics and the capacity of coverage by adding constraints to the velocity space. Moreover, the 2-D environment is demonstrated in this paper; the method can be further extended to the 3-D situation.

## Figures and Tables

**Table 1:** Symbols and their description.

Symbol	Description
$v_{opt}$	The optimal velocity decision.
PR	The permitted region with unknown shape.
Square(x)	Centered at 0, the length of edge is twice that of x.
RV(S)	Random velocity in the set of S.
AVE(G)	The center of the set of G in Euclidean Space.
IdleVel()	The velocity of 0
NUM(S)	The number of point in the set of S.

**Table 2:** Simulation parameters.

Parameter	Value	Description
T	0.25 s	simulation step size
ζ	$10^{-5}$	algorithm terminated value
$l_{e}$, $l_{a}$	250 m, 50 m	length of square region $\Omega_{e}$, $\Omega_{a}$
n, $n^{\prime }$	25, 1000	number of UAVs
$v_{i}^{\max}$	2 m/s	maximum velocity of UAVs
r, R, CR	0.5 m, 25 m, 70 m	radius of UAVs’ shape, sensor and communication
$n_{i}^{\max}$, $R_{i}^{\max}$	4, 51 m	maximum considered neighbor and distance

**Table 3:** Comparison of the calculation speed of UAVs’ scale (n=25).

Method	Case 1	Case 2	Case 3	Ave Time (ms)
RD	10.857	20.280	13.266	14.807
V-Based	591.148	605.856	632.880	609.961
VFA	36.749	48.072	43.573	42.798
